# Proliferation/Quiescence: When to start? Where to stop? What to stock?

**DOI:** 10.1186/1747-1028-6-20

**Published:** 2011-12-09

**Authors:** Bertrand Daignan-Fornier, Isabelle Sagot

**Affiliations:** 1 Univ. Bordeaux, IBGC, UMR 5095, F-33000 Bordeaux, France; 2 CNRS, IBGC, UMR 5095, F-33000 Bordeaux, France

**Keywords:** Quiescence, *Saccharomyces cerevisiae*, Start point, Restriction point, starvation, cell cycle, metabolism

## Abstract

The cell cycle is a tightly controlled series of events that ultimately lead to cell division. The literature deciphering the molecular processes involved in regulating the consecutive cell cycle steps is colossal. By contrast, much less is known about non-dividing cellular states, even if they concern the vast majority of cells, from prokaryotes to multi-cellular organisms. Indeed, cells decide to enter the division cycle only if conditions are favourable. Otherwise they may enter quiescence, a reversible non-dividing cellular state. Recent studies in yeast have shed new light on the transition between proliferation and quiescence, re-questioning the notion of cell cycle commitment. They also indicate a predominant role for cellular metabolic status as a major regulator of quiescence establishment and exit. Additionally, a growing body of evidence indicates that environmental conditions, and notably the availability of various nutrients, by impinging on specific metabolic routes, directly regulate specific cellular re-organization that occurs upon proliferation/quiescence transitions.

## Background

The restriction point is defined as a point in G1 phase of the cell cycle after which cells are committed to cell division [1]. Indeed, it is commonly accepted that once cells have passed this point, they proceed through all the phases of the cell cycle until completion, *i.e*. until they reach the restriction point again. But cells may individually "decide" to engage themselves in another round of cell division or enter a non-dividing state. This non-dividing state may be non-reversible (senescence, apoptosis...) or, alternatively, cells can enter quiescence, a cellular state defined as a temporary and reversible absence of proliferation. In all cases, it is thought that cells integrate a combination of external and internal signals before committing to the cell division cycle. In multi-cellular eukaryotes, external signals emanate from the entire organism. These extremely complex physiological conditions are difficult to reproduce in a laboratory. By contrast, in single cell eukaryotes like budding yeast, these external cues can be easily monitored since entry into-and exit from the cell cycle apparently rely solely on nutrient availability in the growth medium. As mammalian cells, yeast cells, once they have passed a point called Start (originally defined as a point in G1 after which cells are resistant to mating pheromone [2]) are committed to proceed through all the phases of the cell cycle until G1 [3].

The molecular nature of Start has been extensively investigated and a fair amount of key proteins, including cyclins and cyclin-dependent kinases, together with crucial signalling networks have been discovered. However, in yeast, as in metazoa, how these regulators integrate external and internal signals to trigger either the re-entry into the cell cycle or the transition to non-dividing cellular states remains largely mysterious.

Recently, using budding yeast as a model organism, transitions from proliferation to quiescence have been revisited by means of several powerful "omic" approaches and at the individual cell level [4-7]. These studies have re-questioned the existence of a quiescence program, an issue that we have recently discussed elsewhere [8]. Importantly, these studies, centered on quiescence, shed new light on the notion of Start, cell cycle commitment and cell cycle progression.

## Discussion

### When to start?

In his original "Restriction point" paper [1], Pardee set out to "provide evidence that cells that have reached quiescence by a variety of means are indeed in the same state". The idea of a unique quiescent state is still under debate [6,8], yet, Pardee's study concluded that his "results are consistent with the existence of a single switching point, the restriction point in G1, that regulates the re-entry of cell into a new round of the cell cycle" [1]. Now, more than 35 years after this initial proposal, the notion of a restriction point is widely accepted. In fact, the restriction point is often viewed as a sort of fence that cells have to "jump" to enter a new cell cycle. In yeast, elegant studies have clearly demonstrated the irreversible nature of Start, this irreversibility relying on complex transcriptional feedback loops between cyclins, transcriptional repressors and activators [9,10]. Start can therefore be envisioned as a molecular switch. The existence of such a switch was clearly established in specific genetic contexts allowing tight control of cell cycle regulators. However, it is not yet clear whether in a wild type situation, when external conditions are not favourable, all cells do indeed arrest synchronously just prior to start. In fact, it was proposed that upon entry into quiescence, mammalians cells do not necessarily arrest synchronously just at the restriction point, but rather form a cohort of cells arrested as a continuum throughout G1 [11,12]. The possibility of such a non-synchronous arrest remains to be analyzed in close details in yeast. Yet, it becomes more and more clear that, although very helpful to decipher the highly complex network of proteins involved in cell cycle regulation, the simplistic "fence model" most probably does not reflect what happens in a physiological context, where both internal and external signals need to be integrated for the decision to enter or not a new round of cell division.

In the textbooks, the decision to enter a new division cycle is described as an irreversible commitment to an entire cell division cycle; and supposedly once cells have passed start, they HAVE to go through all the successive cell cycle stages back to G1. However, recently we have shown that wild type yeast cells can enter quiescence in other cell cycle stages than G1. In other words, cells may not be committed to cell cycle completion [7]. In fact, using conditional cell cycle mutants, it was found that cells arrested in other cell stages than G1 and then starved, could acquire a property of quiescent cells, *i.e*. heat resistance [13]. More recently, we have shown that a cell cycle mutant arrested in G2/M can assemble structures that are specific to quiescent cells upon carbon source exhaustion. Therefore cells forced to enter quiescence can do so in other cell cycle phases than G1. Importantly, we have also shown that between 2 to 20% of a wild type cell population can "naturally" enter quiescence in other cell cycle stages than G1 upon carbon source exhaustion [7]. Therefore, the decision in G1 is not "to go through all the cell cycle phases back to G1 or die". Cells that have passed the restriction point are not necessarily committed to continue their progression until they reach G1 again, but eventually may arrest elsewhere. In fact, in yeast, the arrest in G1 phase of the cell cycle is neither necessary nor sufficient for quiescence establishment [7]. This may also be true for mammalian cells as quiescent cells in other cell cycle phases than G1 have been reported in human carcinomas [14] and in various cell lines [15]. Furthermore, because the operational definition of quiescence is "a reversible arrest of proliferation", if cells can enter quiescence elsewhere than in G1, this necessarily means that they are capable of exiting quiescence in other cell cycle stages and give rise to a progeny. Consequently, not only the decision to enter a new round of cell division may occur at various points all along G1, as suggested by Cooper [11], but this decision may occur in other cell cycle phases than G1. Importantly, Coudreuse and Nurse have very recently shown that in *S. pombe*, cells can enter mitosis from inappropriate points in the cell cycle, challenging thus the strict sequential interdependency of the cell cycle phases [16].

### Where to stop?

If cells can enter quiescence in various cell cycle stages, then, why do budding yeast and mammalian cells preferentially do so in G1? Could it be that quiescence establishment in G1 provides a selective advantage? In agreement with this hypothesis, we have shown that in *S. cerevisiae*, the ability to give rise to progeny of quiescent cells arrested in other phases than G1 is somehow diminished compared to those arrested in G1 [7]. Alternatively, the arrest in G1 could simply be a passive consequence of a metabolic slow down. Indeed, in yeast, like in mammals, cell growth is not a continuum but predominantly takes place in G1 [17-19]. Furthermore, limiting protein synthesis extends the G1 phase duration, but has little effects on the length other cell cycle stages [20-24]. Therefore, G1 should be the phase encompassing the large majority of the cell population when nutritional conditions are limiting for protein synthesis. Consequently, in *S. cerevisiae*, the fact that quiescence entry mostly occurs in G1 could be the passive result of a metabolic slow down rather than a cell cycle controlled event. As most of the cell growth occurs in G1, a metabolic slow down would predominantly impinge on G1 progression but not on the remaining cell cycle phases. In other words, there could be no need to terminate the cell cycle in G1, but the G1 arrest could only be a consequence of the fact that translation should be most highly active in G1. Yet, this does not exclude the possibility that an arrest in G1 might be more favourable and could have been selected during evolution. However, it has been reported that other organisms such as *S. pombe *[25], *C. neoformans *[26], *T. pyriformis *[27], can enter quiescence in G1 or in G2 depending on the environmental conditions. This diversity could reflect different evolutionary strategies to optimize survival in quiescence.

### What to stock?

If the decision to enter the cell cycle mostly relies on metabolic activity, one could speculate that quiescent cells may have a specific metabolomic signature. Recent global analyses of the metabolome of cells that have entered quiescence by different routes did not reveal any obvious key molecules or any specific metabolic signature of quiescence [6]. In fact, there are more similarities between the metabolome of cells growing extremely slowly because of a limiting amount of a specific nutrient and that of quiescent cells that have been starved for the same nutrient, rather than between the metabolome of cells rendered quiescent by different routes [6]. As proposed by Broach and co-workers, quiescence may be an extreme form of slow growth [6]. Therefore one can finally wonder "what are" quiescent cells specific features? Transcription profiles studies have shown that quiescent cells transcriptome is clearly different from that of G1 arrested cells [28-30]. Furthermore, the profiles are very different depending on the way to enter quiescence, very few genes responding similarly in all the conditions tested [5,6,31,32]. Thus, transcriptional response mostly reflects an adaptation to nutrient limitation rather than a common re-programming that would commit cells to the quiescent state. Even if no "quiescence program" has been deciphered yet, would it be possible to find any cellular properties that are unique to quiescence?

About 50 years ago, Yotsuyanagi observed that mitochondria, while tubular in actively proliferating cells, are entirely reorganized into a cortical vesicular network in non dividing yeast cells [33]. More recently, we have shown that upon carbon source exhaustion, budding yeast cells remodel their actin cytoskeleton and assemble typical actin structures called Actin Bodies [34]. Similarly, we have shown that the proteasome, while localized diffusely in the nucleus of dividing cells is reorganized into cytoplasmic structures, named proteasome storage granules (PSGs) upon carbon source exhaustion [35]. The assembly of these specific structures upon entry into quiescence may be the sign of an ultra-structural commitment to the quiescent state. In fact, in yeast, P-bodies, cytoplasmic structures involved in mRNA degradation, are known to assemble upon quiescence establishment [36,37]. More global analyses based on a GFP fusion library have shown that a large number of diffuse cytoplasmic metabolic enzymes such as Ade4, the phosphoribosylpyrophosphate amidotransferase, are restructured into cytoplasmic granules when proliferation ceases upon nutritional deprivation [38,39]. Similarly, some cytoplasmic chaperones are restructured into foci in quiescent yeast cells [38,40]. Very interestingly, diffuse cytosolic proteins such as the CTP synthase Ura7/Ura8 have been shown to assemble into filaments upon carbon source exhaustion [39]. Further, in quiescent cells, chromatin compaction is increased thanks to the activity of the H1 Histone Hho1 [41]. Most of these reorganizations have been observed upon carbon source exhaustion but do they depend on a unique signal? Do these reorganizations take place if entry into quiescence occurs via other routes? In other words are these reorganizations specific to quiescence or do they respond to specific nutritional signals that are concomitant to quiescence entry?

In the case of Actin Bodies and PSG formation, it has been clearly shown that it does not depend on cell cycle regulated processes since they can occur at all cell cycle stages. Furthermore, while these structures do not form upon exhaustion of other nutrients than carbon, they do form upon acute transfer into water, a situation that may be closely related to what may happen in the wild. While we do not yet know the exact nature of the signal(s) that triggers Actin Bodies and PSG formation, it is clear that the disassembly of both structures is caused by addition of glucose [7]. Other structures are not dependent on carbon source availability but rather depend on the concentration of a specific metabolite of their own metabolic pathway. For example, in the case or the CTP synthase filaments, it seems that their formation is conditioned by the availability of triphosphate nucleotides (NTPs), the CTP synthase end products. Indeed, most NTPs inhibit the enzyme activity and cause its polymerization into cytoplasmic filaments [39]. Similarly, Ade4 foci formation is regulated by the presence of adenine in the medium. However, it is interesting to note that by contrast to CTP synthase, Ade4 foci form when the enzyme is active. This led the authors to propose the idea that foci formation may enhance substrate channelling and facilitate metabolite flux control upon nutritional stress [38]. Therefore, specific signals may trigger specific reorganizations, independently of quiescence establishment. Moreover, different signals can lead to the assembly of similar structures. Indeed, P-bodies formation can be observed both upon carbon source exhaustion and upon various cellular stresses [36,37]. In fact, some reorganization may even be passive. For example, the increase in the association of Hho1 with chromatin which causes DNA compaction upon carbon source exhaustion, is apparently not due to an active regulatory process, but rather reflects the reduction of Hho1 displacement by RNA polymerase II as a result of low expression of most genes in quiescent cells [41]. In light of these data, we propose that reorganizations occurring in quiescent cells depend on the cell's needs and are triggered on a "on demand" basis. This hypothesis fits with the fact that cell's gene expression and metabolic cartographies are drastically different depending on the nature of the exhausted nutrient [6]. In fact, cells may stock the unutilized proteins, as it is probably the case with the actin embedded in Actin Bodies. Why would yeast cells make reserves of unused proteins instead of degrading them? Degradation would require the adequate recognition and the active proteolysis of myriad unused proteins that are neither damaged nor misfolded. Instead, stocks could either be used to face some sporadic stresses during quiescence and/or improve fitness upon exit from quiescence and re-entry into the proliferation cycle.

## Conclusions

Quiescence is at the heart of two major processes in biology: controlling the cell proliferation and facing chronological aging. Astonishingly little is known about this cellular state and the research field needs new tools to reveal the molecular processes that regulate transitions between cell cycle progression and quiescence. There is a growing number of examples of cellular reorganizations upon entry into quiescence and probably more to discover. May these structures be used as markers of quiescence? While their universality is questionable, just as is the existence of a unique quiescent state, these structures enable us to work at the individual cell level and are therefore useful to comprehend how cells solve the Cornelian dilemma of dividing or not!

## Abbreviations

PSG: Proteasome Storage Granules; NTPs: tri-phosphate nucleotides.

## Competing interests

The authors declare that they have no competing interests.

## Authors' contributions

I.S. and B.D.-F wrote the manuscript. All authors have read and approved the final manuscript
